# Development of Cerebral Metastasis after Medical and Surgical Treatment of Anal Squamous Cell Carcinoma

**DOI:** 10.1155/2012/912178

**Published:** 2012-10-09

**Authors:** Andrew Austin Gassman, Emil Fernando, Casey Jacob Holmes, Umesh Kapur, Joshua M. Eberhardt

**Affiliations:** ^1^Division of Colorectal Surgery, Department of Surgery, Loyola University Medical Center, Maywood, IL 60153, USA; ^2^Stritch School of Medicine, Loyola University Medical Center, Maywood, IL 60153, USA; ^3^Department of Pathology, Loyola University Medical Center, Maywood, IL 60153, USA

## Abstract

Squamous cell carcinoma of the anus is a relatively uncommon GI malignancy. When it does occur, it metastasizes in only a small minority of patients. Spread of anal squamous cell carcinoma to the brain is exceedingly rare, and has been previously reported only three times in the medical literature. We report the case of a 67 year old male who was diagnosed on presentation with a poorly differentiated anal squamous cell carcinoma that already had a solitary metastasis to the liver. While the tumors were initially responsive to chemoradiotherapy, the patient's primary and liver lesions recurred. The patient then underwent synchronous abdominoperineal resection for the primary lesion and a liver lobectomy for the metastasis. Soon thereafter, the patient developed focal neurologic symptoms and was found to have an intracranial lesion that on biopsy demonstrated metastatic squamous cell carcinoma. This case highlights the fact that patients with a previous history of anal squamous cell carcinoma can occasionally develop cerebral metastasis. Furthermore, cerebral metastases from anal squamous cell carcinoma portend a dismal prognosis even in the face of aggressive medical and surgical therapy.

## 1. Introduction

Anal carcinomas represent approximately 4% of gastrointestinal cancers diagnosed annually [1]. The majority of anal carcinomas are squamous cell carcinomas (SCCs) for which the treatment has been well-established and the prognosis is usually favorable [2]. The rate of metastatic disease is low and is typical to either the liver or the lungs. Here we report a case of primary anal SCC that presented with a single isolated hepatic metastasis and fatal cerebral metastasis shortly thereafter. Cerebral metastasis from anal SCC is quite rare, with only three other cases being reported in the literature [3–5].

## 2. Case Presentation

**Figure 1:**
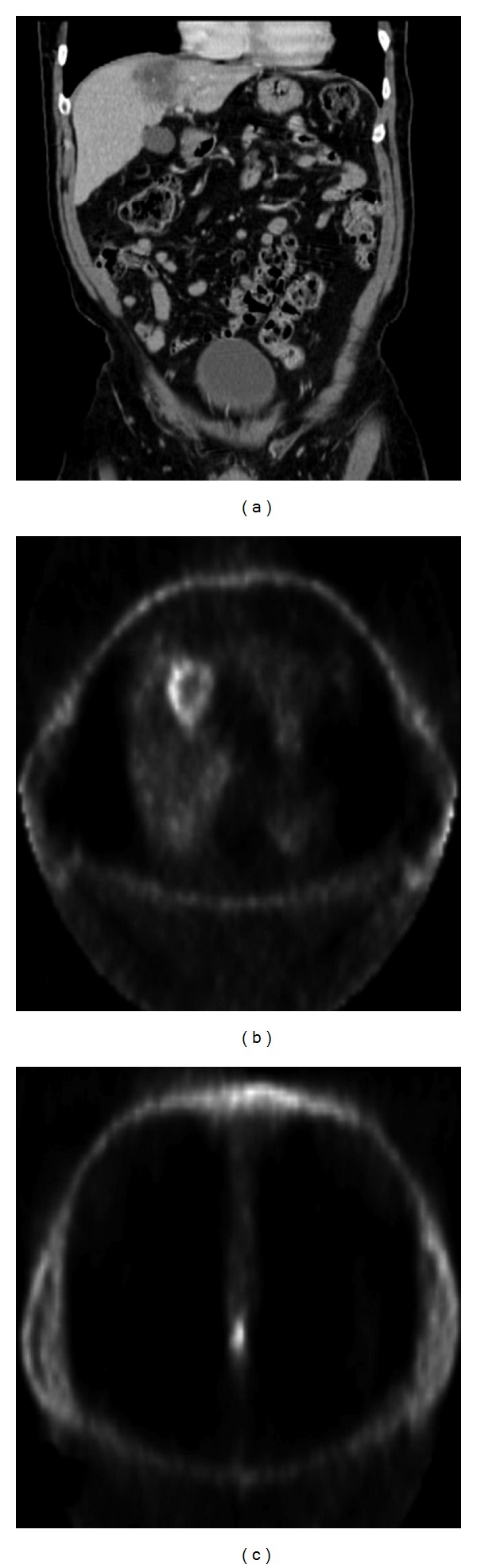
CT (a) and PET (b) showing hepatic metastasis, and (c) PET showing anal primary.

**Figure 2:**
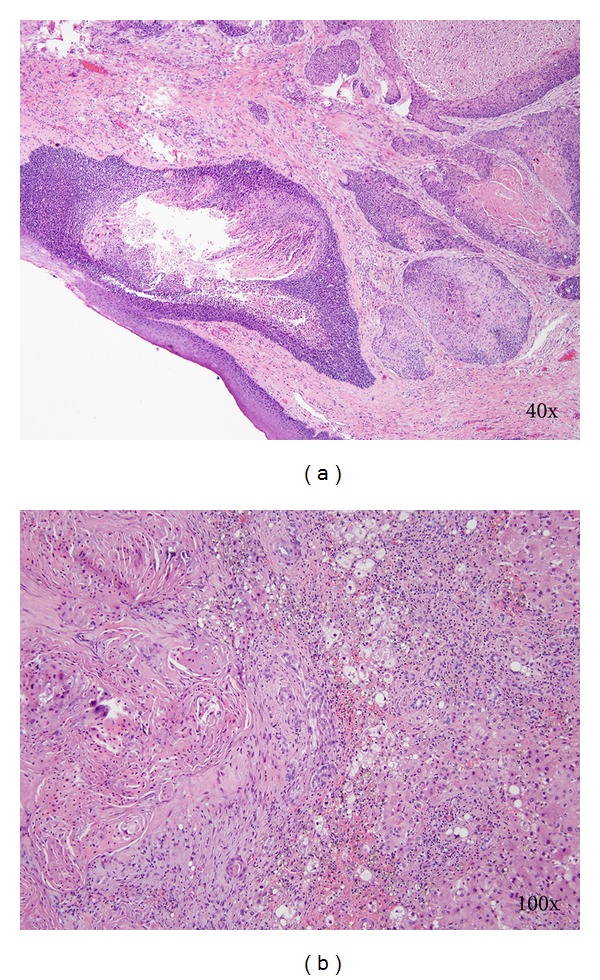
Histologic sections showing squamous cell carcinoma in the APR specimen (a) and the liver resection (b).

A 67-year-old male with chronic pulmonary and cardiac conditions developed blood per rectum and a change in stool caliber which lead to workup with colonoscopy. This demonstrated a 4 cm proximal anal canal lesion with biopsy identifying invasive poorly differentiated squamous cell carcinoma. Staging CT of the brain, chest, abdomen, and pelvis demonstrated an isolated hepatic metastasis as the only distant site of disease (Figure 1). The patient was treated with chemoradiotherapy including 5-FU, Cisplatin, and 50.4 Gy radiation followed by further Cisplatin-based systemic therapy. Twelve weeks after completion of chemoradiotherapy, anoscopy demonstrated complete resolution of the anal canal lesion with a remaining scar that was biopsied and was negative for carcinoma. Additionally, imaging of the liver revealed reduction in the size of the hepatic metastasis. However, 6 months later, surveillance anoscopy and imaging revealed recurrence of the anal canal primary and enlargement of the hepatic metastasis with biopsies of both demonstrating SCC. The anal lesion was increasingly symptomatic causing pain, tenesmus, and bleeding. Repeated imaging of the brain, chest, abdomen, and pelvis confirmed that the disease was still isolated to the liver and anal canal; therefore, synchronous abdominoperineal and liver resection were undertaken. Pathologic analysis revealed that the primary was 5.7 cm and was associated with negative lymph nodes, proximal, distal, and radial margins, and the hepatic metastasis was 6.1 cm and also had negative resection margins (Figure 2).

**Figure 3:**
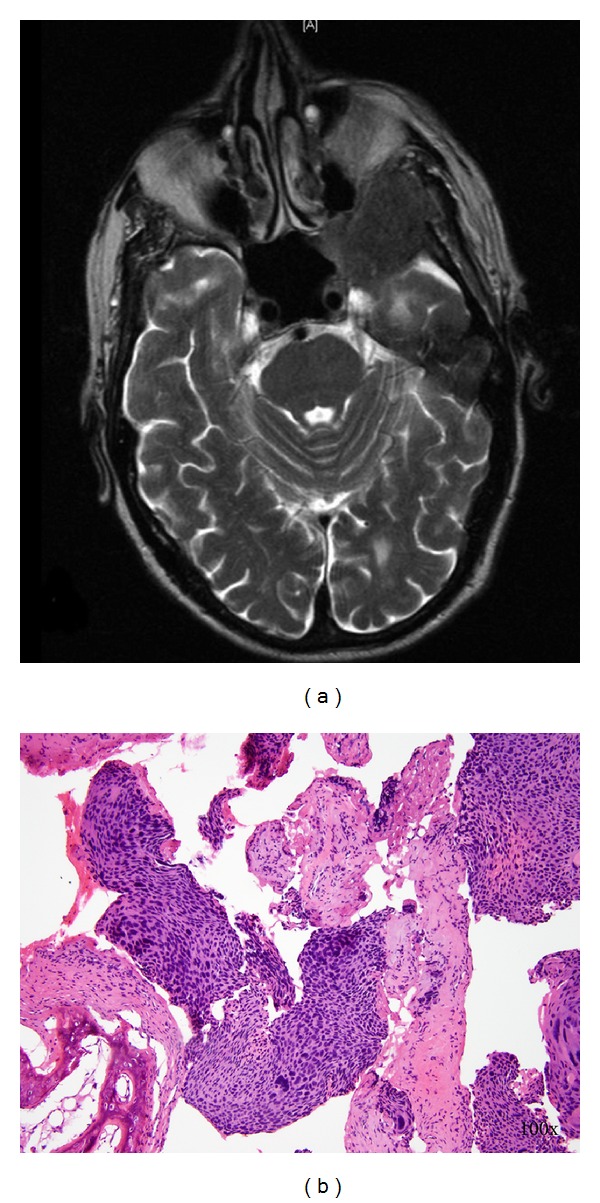
MRI demonstrating left frontal intracranial lesion (a) and histology from the biopsy of this lesion showing metastatic squamous cell carcinoma (b).

Despite having an uneventful postoperative course, three months after surgery, the patient presented with blurry vision and right eyelid ptosis. CT of the head and MRI of the brain demonstrated a 4 cm intracranial mass eroding the sphenoid and temporal bones, and an involvement of the left orbit, optic nerve, and rectus muscle (Figure 3). Biopsy demonstrated metastatic SCC identical to the anal and hepatic lesions (Figure 3). At this point stereotactic radiotherapy was planned however the patient succumbed to his disease prior to starting this therapy.

## 3. Discussion

Squamous cell carcinoma of the anus comprises about 75% of all anal malignancies with a male-to-female ratio of 1 : 1.5 [3]. It arises most commonly from the squamous epithelium in the distal half of the anal canal, with a minority originating from the transitional zone mucosa [6]. Although it currently comprises only approximately 4% of gastrointestinal tract malignancies, the incidence is slowly increasing; current data shows the incidence to be 0.7 per 100,000 per year [1, 2, 6, 7]. The exact reason for this increase is unknown, but hypotheses include increases in the number of patients taking immune-suppressing medications for organ transplants or other disorders, and an increase in immune-suppressing diseases such as HIV/AIDS [1]. Other risk factors known to be associated with the development of anal SCC include smoking, history of sexually transmitted disease, and history of anogenital human papilloma virus infection [6]. In fact, some studies have shown that HPV DNA, especially types 16 and 18, is present in up to 88% of anal SCC [6]. Furthermore, recent work using vaccines that target these viral serotypes has shown some risk reduction in the development of precursor lesions [8, 9]. 

Since the original description by Nigro et al. [10], chemoradiation has become the primary treatment modality for anal canal SCC. It has been shown to provide equivalent local control and survival rates as abdominoperineal resection without the major morbidity and mortality associated with the surgery [7, 10]. Additionally, randomized control trials performed by multiple groups have shown the superiority of chemoradiotherapy over radiotherapy alone [11, 12]. Current chemoradiotherapy treatment protocols, as used in our case, have been shown to result in 5-year survival rates ranging from 65% to 90% depending on the series [1, 6, 13]. 

Patients with persistence or recurrence of anal SCC after chemoradiation represent a small but particularly challenging group. Failure of clinical response to chemoradiation occurs in 10%–15% of patients, while recurrence occurs in 10%–30% [14]. Under these circumstances, salvage resection can be considered, and when the disease is isolated to the pelvis, salvage abdominoperineal resection has been shown to result in 5-year survival rates of 24%–47% [15–19]. However, salvage surgery in this setting is also associated with local recurrence rates as high as 86% [19].

Treatment of metastatic anal canal SCC has no clear consensus. In our case, both the primary and the hepatic metastasis responded after initial chemoradiotherapy, and systemic chemotherapy, but this was followed shortly thereafter by failure. Given the symptomatic nature of the primary and the fact that the disease was resectable and limited to the pelvis and liver, we felt surgery was a reasonable option. In fact, previous studies have shown the efficacy of hepatic metastectomy for anal SCC. Pawlik et al. [20] also reported on 27 patients undergoing hepatic resection for metastatic anal SCC of which 88% had previously received multimodality therapy [20]. Additionally, they found that hepatic resection was associated with a 9.6-month disease-free survival and 5-year overall survival of 22.9%. Pawlik also found that hepatic tumor >5 cm and positive surgical margins were independent risk factors for poorer outcome.

Only three prior cases of anal SCC metastatic to the brain have been reported [3–5]. The 2011 report from Rughani et al. described a patient with anal SCC presenting with hepatic metastasis which was initially treated with chemoradiotherapy. Soon after completion, the patient developed neurologic symptoms and was found to have a cerebral mass that underwent further treatment including surgery and radiation. The patient succumbed 14 weeks after discovery of the cerebral metastasis [3]. In a large review of nearly four hundred anal cancers reported by Klotz et al., only one case was identified where brain metastasis had occurred [4]. Unfortunately details were not given with regard to that particular patient's treatment or overall outcome. Davidson and Yong reported a case where a patient, who had previously undergone an abdominal peritoneal resection for anal cancer, developed neurologic symptoms eight years after the surgery and was found to have a brain lesion whose pathology was consistent with a metastasis from the original primary [5]. The patient was treated with craniotomy, resection, and radiotherapy, and in the followup period developed large recurrence of the primary tumor.

## 4. Conclusions

Cerebral metastasis from anal SCC is extremely rare, and this case is now the fourth to be reported in the literature. Taking all reported cases together, it is apparent that cerebral metastasis is associated with a poor prognosis, even with aggressive medical and surgical therapy. Treatment with palliative intent alone might be most justified in these cases. Additionally, cerebral metastasis may present in a delayed fashion, even years after successful treatment of the primary. Therefore, the diagnosis should be entertained when patients with a history of this malignancy present with new neurologic symptoms.
